# Exercises for Women with Persistent Pelvic and Low Back Pain after Pregnancy

**DOI:** 10.5539/gjhs.v8n9p107

**Published:** 2015-10-29

**Authors:** Monica Unsgaard-Tøndel, Ottar Vasseljen, Astrid Woodhouse, Siv Mørkved

**Affiliations:** 1 Sør-Trøndelag University College, Faculty of Health and Social Science, Department of Physiotherapy, Trondheim, Norway; 2 Norwegian University of Science and Technology, Faculty of Medicine, Department of Public Health and General Practice, Trondheim, Norway; 3 Department of Cancer Research and Molecular Medicine, Trondheim, Norway

**Keywords:** Transversus abdominis, pregnancy, low back pain, pelvic pain, ultrasound, exercises

## Abstract

**Background:**

Specific stabilizing exercises activating deep local muscles in coordination with global muscles are recommended in the treatment of pregnancy-related lumbopelvic pain. Some studies have suggested that recruitment of the deepest abdominal muscle, transversus abdominis, is crucial in the development and improvement of lumbopelvic pain.

**Objective:**

This exploratory study aimed to describe the development of pain, disability and transversus abdominis recruitment before, during and after an individually designed intervention including an exercise program for women with persisting lumbopelvic pain after delivery.

**Design:**

A multiple-baseline, single-subject experimental design was applied.

**Methods:**

Sixteen women with lumbopelvic pain after delivery were included and received tailored exercise therapy, including ultrasound-guided activation of deep muscles, strengthening and stretching exercises and advice. Pain, disability and ultrasound-recorded activation of transversus abdominis was registered weekly. Treatment and testing was performed in a primary care setting in Trondheim, Norway.

**Results:**

All sixteen included women reported reduced pain and decreased disability over the intervention period. The magnitude of transversus abdominis activation varied substantially between individuals and tests. While there was a statistically significant correlation between change in pain and change in disability, no correlation was observed between change in transversus abdominis activation and change in symptoms.

**Limitations:**

This is an exploratory study and results cannot be generalized without replication in controlled studies.

**Conclusions:**

Pain and disability due to persistent low back and pelvic pain after delivery were reduced after specific, individual adapted exercise including deep and superficial lumbopelvic muscles. Changes in pain and disability were not associated with changes in transversus abdominis activation.

## 1. Introduction

Lumbopelvic pain is a common complaint during and after pregnancy. A systematic review found that 44% of pregnant women and 25% of newly delivered women experienced low back- and/or pelvic pain (Wu et al., 2004). One out of three in a Swedish population of newly delivered women reported lumbopelvic pain 3 months after delivery (Gutke, Lundberg, Ostgaard, & Oberg, 2011). A Norwegian study found that in 16% of women reporting pelvic pain during pregnancy, pain persisted 3-6 months after delivery (Gausel et al., 2015). For some, pain-related reduction of physical activity as well as diminished quality of life may persist several years after delivery. Consequently, pregnancy-related low back- and pelvic pain represents an extensive problem, both for individual women, families, and for society.

The cause of pregnancy-related lumbopelvic pain is acknowledged as multifactorial, and related to the dynamic stability of the pelvis (Vleeming, Albert, Ostgaard, Sturesson & Stuge, 2008). During pregnancy, hormonally induced increase of ligament laxity leads to a slightly larger range of movement in the pelvic joints (Mens, Pool-Goudzwaard, & Stam, 2009). Biomechanical studies (Vleeming, Schuenke, Danneels, & Willard, 2014) have indicated that this decreased joint stability may be compensated for by enhanced deep local muscle activation. The innermost abdominal muscle, transversus abdominis, has been demonstrated to increase sacroiliac joint stiffness (Richardson et al., 2002). Based on results from randomised controlled trials, guided exercises specifically targeting both the local and global muscles have been recommended in European guidelines (Vleeming et al., 2008). These trials have typically included a mixture of exercises including isolated deep abdominal muscle activation, however without measuring changes in the muscles targeted. Additionally, though isolated deep muscle activation has been recommended, little is known about the relation between deep muscle recruitment and symptom relief. Therefore, which of the exercise elements are the most effective and whether transversus abdominis should be specifically targeted remains unknown. Additionally, the importance of tailoring interventions aiming at each patient’s unique problems and clinical findings is increasingly acknowledged. Such interventions require knowledge of the specific factors and processes affecting individual response to therapy. Single subject experimental design allows for scrutiny of changes in the targeted outcomes for individual patients throughout the whole intervention period (Portney, 2014). Consequently, this design is suitable for continuous observation of associations between symptoms and muscle activation during individually tailored exercise therapy. A single case study have reported parallel symptom relief and improvement in transversus abdominis activation during biofeedback training in one patient with pregnancy related pelvic pain after delivery (Rajalakshmi & Senthil Kumar, 2012). There is a need of more studies describing development of symptoms and muscle activation before, during and after such specific exercises.

### 1.1 Aim

This study aimed to explore the week-to-week development of pain intensity and disability in 16 women with persistent pregnancy-related lumbopelvic pain the during an individually adapted exercise program. A secondary aim was to explore correlations between changes in disability, pain intensity, and voluntary activation of transversus abdominis (the deepest abdominal muscle) observed by ultrasound.

## 2. Methods

### 2.1 Design

A multiple-baseline single subject experimental A_1_ B A_2_-design was applied. Phase A_1_ (4 weeks’ duration) represented the baseline phase, phase B (16 weeks duration) the treatment phase, and phase A_2_ (4 weeks duration) constituted the treatment withdrawal phase (Portney, 2014). This design was chosen in order to scrutinize individual response over time and to compare treatment effects on single subjects. The intervention was the independent variable in this study. Dependent variables were patient response in terms of self-reported pain and disability in addition to voluntary activation of transversus abdominis measured by ultrasound imaging. Registrations of the dependent variables were performed weekly.

### 2.2 Participants

Twenty-one participants were recruited from midwifes, physicians, physiotherapists and nurses in maternity-care units at the local university hospital and Trondheim primary healthcare. Included participants had pregnancy-related pain in the lower back and / or pelvic region at least 3 months after delivery and were referred from their general practitioner. Age, parity, delivery method, and clinical examination results were registered initially. Individually adapted exercises were tailored according to the initial findings. Exclusion criteria were: rheumatic diseases, malignancy, central-nervous disease, skeletal anomalies, acute nerve compression, serious cardiovascular disease, back problems where physical stress was contra-indicated, recent surgery, substance abuse, insufficient language capabilities, diagnosed psychiatric disease, and pregnancy. Table 1 presents background variables that may influence improvement potential (Gutke, Ostgaard, & Oberg, 2008). The study was approved by the regional ethics committee and the declaration of Helsinki was followed.

**Table 1:** Participants’ characteristics

	Age	BMI	Work status	Symptom intensity^1^			Pain sites^2^	TrAslide^3^	Delivery		

									Time^4^	Method	Weight^5^
1	34	23	Student	Severe	6.6	5.4	Back	-	36	Vacum	4.5
2	36	27	Sick leave	Severe	3.5	6.2	No	0.8	3	Vaginal	4.4
3	35	25	Work	Moderate	0.9	3.6	No	1.1	24	Vaginal	3.8
4	33	26	Maternity	Severe	3.1	3.8	No	1.0	6	Vaginal	4.8
5	25	20	Maternity	Severe	1.8	2.1	Back	0.5	12	Vaginal	3.7
6	36	24	Work	Severe	2.4	2.0	No	1.3	72	Vaginal	4.2
7	29	32	Maternity	Severe	4.8	3.9	Pelvic	-	3	Sectio	4.6
8	30	21	Maternity	Moderate	5.1	5.2	Hip	1.1	3	Vacum	3.1
9	29	17	Maternity	Severe	3.8	5.2	Back	0.8	8	Vaginal	3.7
10	25	17	Maternity	Severe	5.5	5.1	Back	0.8	8	Vaginal	3.2
11	27	25	Maternity	Severe	3.2	4.5	Back	1.1	12	Vaginal	3.0
12	30	29	Maternity	Severe	1.6	4.3	Back	1.6	6	Vaginal	4.0
13	28	30	Maternity	Severe	1.2	2.2	No	0.8	3	Sectio	1.2
14	29	26	Work	Severe	6.1	4.5	No	0.8	30	Vacum	3.8
15	29	26	Maternity	Severe	5.9	4.6	Back	1.3	6	Vacum	3.2
16	34	24	Maternity	Moderate	1.8	0.5	No	0.9	3	Vaginal	4.0

1 Participant-reported symptom intensity. Left column: Pain in pregnancy. The middle column: Pain at inclusion. Right column: Disability at inclusion. Responses for middle and right columns were scored on a 0-10 scale, 0 = no pain, 10 = worst.

2 Pain location for participants that had experienced musculoskeletal pain before pregnancy.

3 Measured in centimeters. Average value left and right side, slide in centimeters during abdominal drawing in manuevre.

4 Months since delivery.

5 Kg.

### 2.3 Outcome

Outcome measures were performed once a week during 4 weeks for initial baseline, 16 weeks of treatment and 4 weeks for treatment withdrawal phase.

#### 2.3.1 Primary Outcome

Primary outcome measures were pain and disability. Pain intensity was measured by the 0-100 millimeter Visual Analogue Scale ranging from 0 (no pain) to 100 (maximal pain intensity) (Grotle, Brox, & Vollestad, 2004). Disability was measured by Disability Rating Index (0-100). These instruments have demonstrated responsiveness for changes in disability and pain in patients with long-lasting low back pain. Measurement error reported as minimal detectable change has been reported to be acceptable (17.6 points) for the Disability Rating Index in patients with pelvic girdle pain (Grotle, Garratt, Krogstad Jenssen, & Stuge, 2012). The minimal clinical significant change for the Visual Analogue Scale has been suggested to be 15 points (Ostelo et al., 2008).

#### 2.3.2 Secondary Outcome

**Figure 1:**
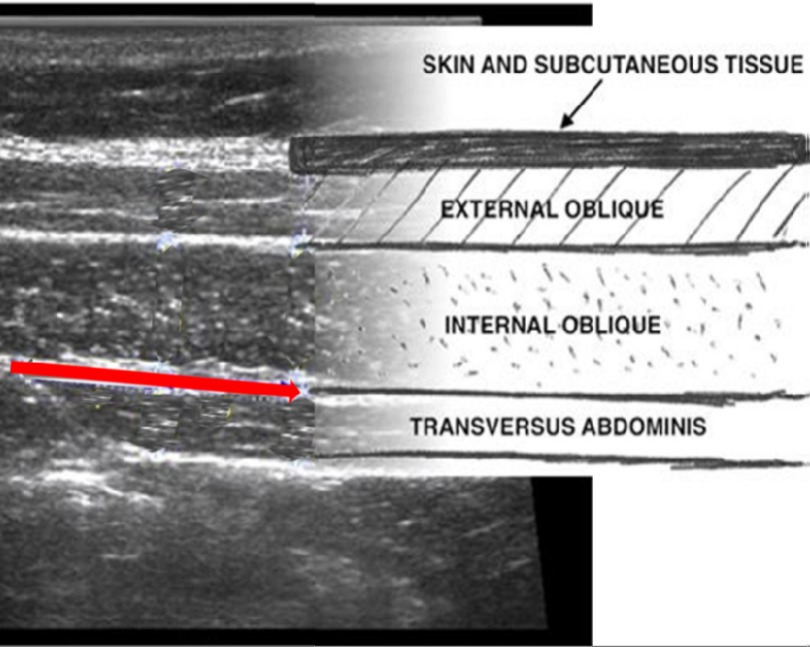
Ultrasound recordings procedure

Ultrasound imaging of displacement and cross-section in deep abdominal muscles (Unsgaard-Tøndel, Lund Nilsen, Magnussen, & Vasseljen, 2012) was also registered before and after intervention. Voluntary deep abdominal muscle activation during abdominal drawing-in maneuvre was measured by brightness-mode ultrasound imaging to evaluate local motor activation. Ultrasound recordings were performed by a moderately experienced operator. Ultrasound recordings were made with a Vivid 7 ultrasound scanner with a M12 linear transducer set to 10 MHz (GE-Vingmed Ultrasound, Horten, Norway). The ultrasound transducer was placed transversally, halfway between the 11^th^ costal cartilage and the iliac crest, oriented in an oblique angle approximating the muscle fibre direction of transversus abdominis and adjusted so that the transverse and oblique abdominal muscles were visible on the screen (Figure 1).

Motor performance during the abdominal drawing-in manuevre was evaluated by measuring slide of transversus abdominis’s anterior fascia during the abdominal drawing-in manuevre. This exercise aimed to obtain an isolated, low-load contraction of transversus abdominis, without activity in the superficial abdominal muscles. The participants were instructed to pull the lower abdomen in towards the spine at the end of normal expiration. The contraction was held for 5-10 seconds and performed twice with ultrasound recordings from each side of the abdomen. Transducer location was adjusted so that the v-shaped midline border of transversus abdominis appeared towards one side of the display. Lateral slide of the midline border was measured by scrolling the video to obtain the distance between the resting and contracted position. Measurement of transversus abdominis lateral slide was used as an indicator of tightening of the anterior fascia and an indirect measure assessing the shortening of the muscle following motor activation (Teyhen et al., 2007). Light pressure on the skin was maintained during recordings and care was taken to keep the transducer in constant position to substantiate that architectural change expressed neuromuscular activation.

Transversus abdominis slide was measured by tracking the medial musculofascial junction and measuring its lateral displacement when scrolling the ultrasound video cineloop.

### 2.4 Intervention

The intervention consisted of 16 weekly treatments lasting one hour to ninety minutes. Individually adapted ergonomic advice, relearning strategies to isolate muscle contraction in transversus abdominis, pelvic floor muscles and the deep fibres of m. multifidus (MF), in addition to coordination- and strength exercises performed on a Swiss ball, and stretching exercises was included. Visualisation of muscle activity by ultrasound imaging was used as feedback to aid voluntary activation of transversus abdominis relative to superficial abdominal muscles (Hodges, 2011).

Pelvic floor muscle contractions were instructed and monitored by pressure biofeedback and vaginal palpation. A vaginal balloon catheter, connected to a pressure transducer (Camtech Ltd, 1300 Sandvika, Norway) was used to measure vaginal pressure during pelvic muscle contractions (Morkved, & Bo, 2000). Deep multifidus muscle contractions were taught in and controlled by palpation. The patient was taught how to recruit the deep muscles and reduce unwanted activity of superficial muscles. At first, deep abdominal muscle activation was taught by the following verbal instruction. “Breath in, breath out, now slowly and gently draw your lower abdomen in towards your lower spine. Gently hold this contraction while breathing”. Difference between deep and superficial abdominal muscle activation was demonstrated on the ultrasound screen. Progression was achieved by pre-activating these deep local muscles in dynamic functional tasks. Swiss-ball exercises for stimulation of muscle-tendon-fascia-slings that stabilises the low back-pelvic area combined with stretching exercises were the other main ingredients in the intervention. Manual treatment and dry needling of muscular trigger points was provided when considered suitable. Individual treatment was given once a week and participants were recommended daily performance of individually adapted home exercises.

### 2.5 Analysis

Mean scores on the outcome variables and Pearson r for correlation between score on outcome variables were obtained in SPSS 21. Individual scores were plotted (Figures 3-7) in order to utilize visual inspection of individual patient outcome, week by week. Analysis by visual inspection is a key feature of the single subject experimental design, and allows exploring trends even for variables with considerable natural variability, like pain and pain-related disability (Portney, 2014).

**Figure 2:**
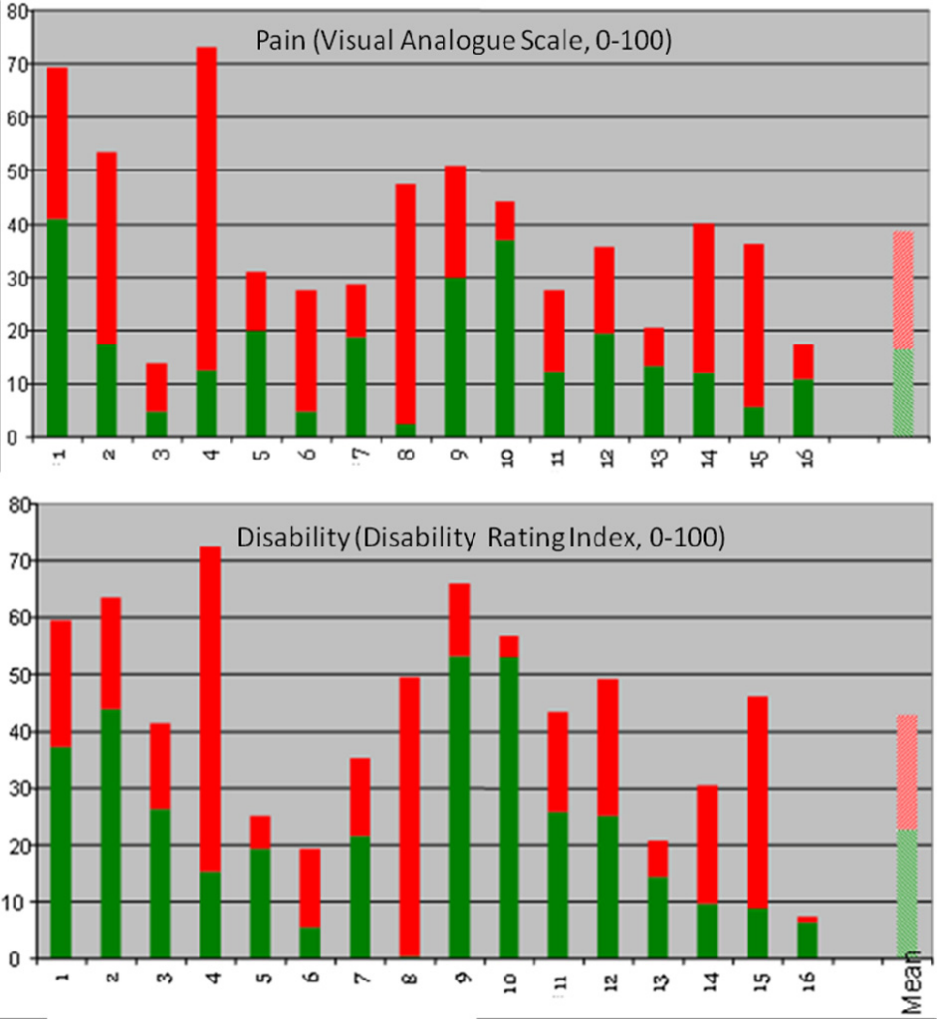
Pain and disability before and after exercise Pain and disability reported before (top of red columns) and after (top of green columns) treatment. Average score for each of the 16 participants over first baseline period (weeks 1-4) and second baseline period, the treatment withdrawal phase (weeks 21-24). The red columns represent the symptom reduction for each individual participant over the treatment period.

**Figure 3:**
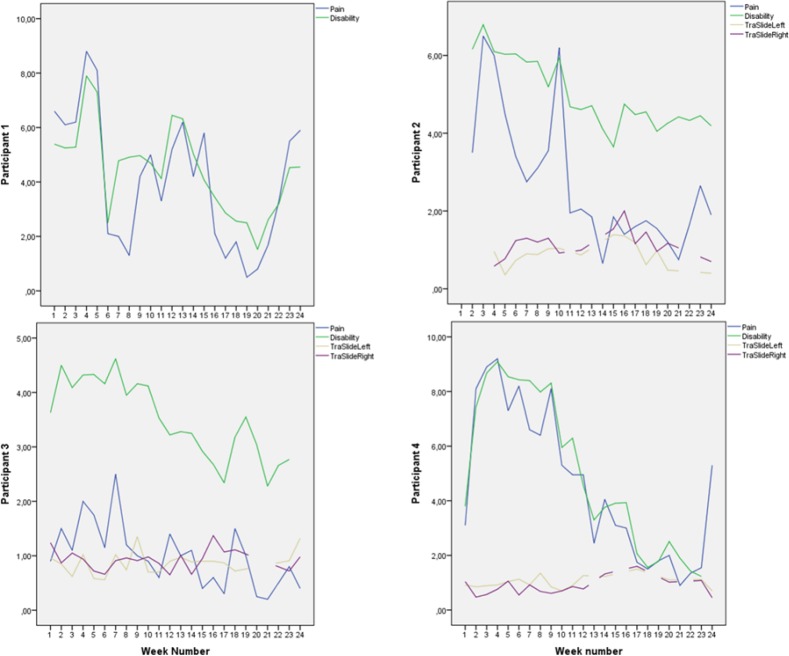
Pain, disability, and deep abdominal muscle activation for participants 1-4, week for week Pain and disability intensity measured in centimeters from zero on the 0-100 mm visual analogue scale. Transversus abdominis slide was also measured in centimeters.

**Figure 4:**
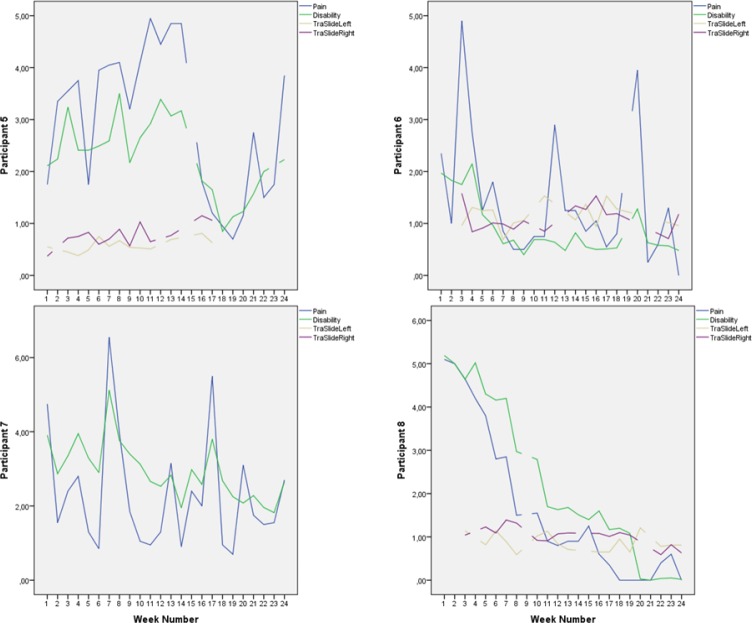
Pain, disability, and deep abdominal muscle activation for participants 5-8, week for week Pain and disability intensity measured in centimeters from zero on the 0-100 mm visual analogue scale. Transversus abdominis slide was also measured in centimeters.

**Figure 5:**
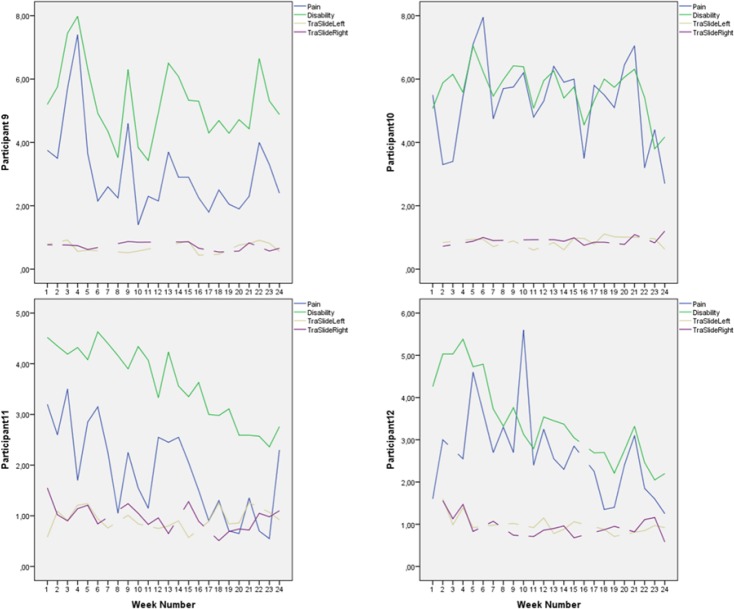
Pain, disability, and deep abdominal muscle activation for participants 9-12, week for week Pain and disability intensity measured in centimeters from zero on the 0-100 mm visual analogue scale. Transversus abdominis slide was also measured in centimeters.

**Figure 6:**
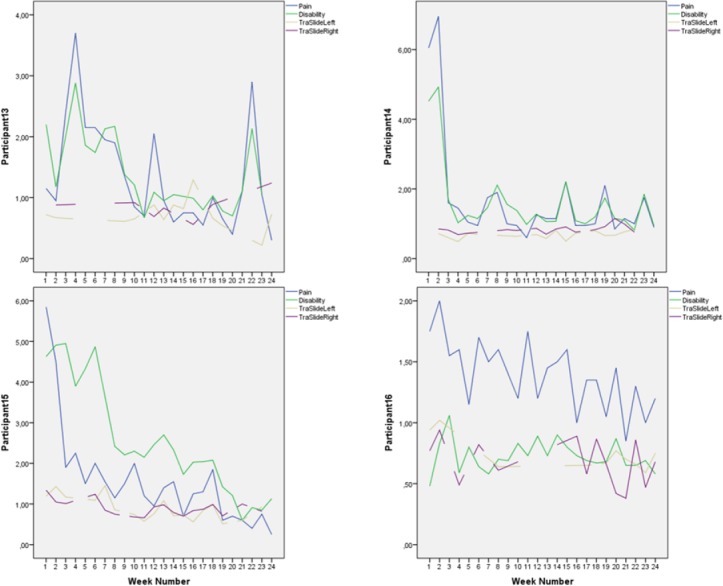
Pain, disability, and deep abdominal muscle activation for participants 13-16, week for week Pain and disability intensity measured in centimeters from zero on the 0-100 mm visual analogue scale. Transversus abdominis slide was also measured in centimeters.

**Figure 7:**
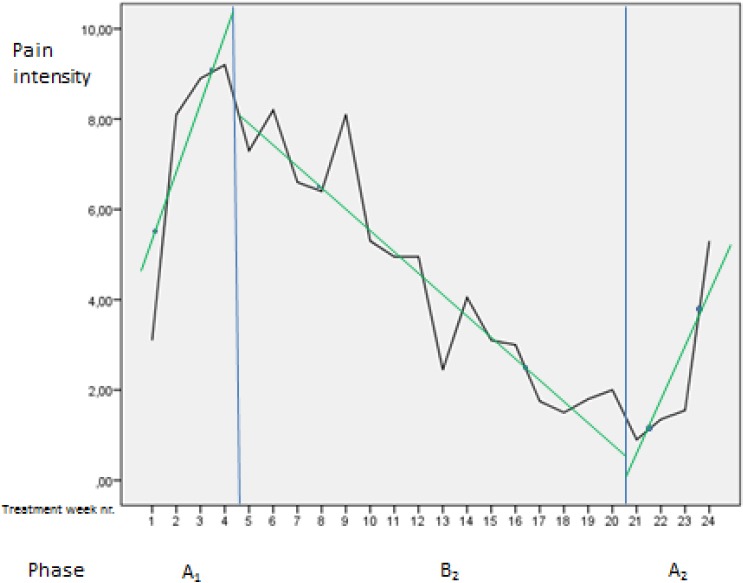
The figure illustrates weekly pain intensity with celeration line for participant 4 Pain intensity was measured in centimeters from zero on the 0-100 mm visual analogue scale.

## 3. Results

A total of 21 persons agreed to participate. Five women were excluded after initial assessment, two because of skeletal anomalies and three because they were unable to comply with the intervention. Sixteen women mean age 31 (range 25-36), parity 1,7 (range 1-4) with persistent low back- and pelvic pain after pregnancy at least 3 (range 3-72) months after the last delivery was included (Table 1).

Mean score on the Disability Rating Index and Visual Analogue Scale for pain intensity was reduced for everyone after the intervention period (Figure 2). Ultrasound measurements of deep abdominal muscle activation demonstrated a substantial variation in transversus abdominis slide between tests and individuals (Figures 3-6). Visual inspection of the graphs and correlation estimates did not suggest an association between transversus abdominis slide and symptoms, or between the magnitude of slide and time / treatment phase.

Mean pain intensity reported for all participants was 38 over the pre-treatment baseline period and 18 over the post-treatment baseline period on the Visual Analogue Scale. Mean disability was 43 over the pre-treatment baseline period and 23 over the post-treatment baseline period, on a similar 0-100 scale. Participants 1, 2, 4, 8, 12, 14, and 15 obtained clinically important reductions both in disability and pain intensity, according to earlier suggested limits (Grotle et al., 2012; Ostelo et al., 2008). Participants 6 and 9 obtained clinically significant pain reduction, but not disability reduction within the earlier defined limits (Figure 2). A tendency towards increased symptoms in the treatment withdrawal phase was observed for participants 1, 4 and 5 (Figures 3 and 4). Significant correlation was found between change in pain and disability over the baseline periods (Pearson’s r = 0.89, p = 0.001).

The largest and most consistent symptom reduction was observed for participants 4, 8, and 15. These participants had delivered only 3-6 months before inclusion. Most of the other participants had pain of longer duration. Except from that, the most successful participants did not seem to differ from the others for the registered background variables (Table 1). Participant 4 experienced some symptom increase during the baseline A_1_ phase, followed by a marked and relatively parallel drop in pain and disability during the 16 weeks of treatment (Figures 3 and 7). Though she experienced some symptom variation from week to week, the celeration line suggests improvement in the treatment phase and stability followed by some pain increase at the end of the withdrawal phase. Some co-variation between symptom reduction and transversus abdominis slide cannot be excluded for this participant. For participants 8 and 15, the data do not suggest that symptom reduction was associated with increased transversus abdominis activation. For these participants, some of the symptom reduction started one or two weeks before the treatment phase. Participant number 8 had the most marked improvement of pain and disability throughout the intervention period (Figures 2 and 4), largely during the first part of the treatment period, while transversus abdominis recruitment did not change markedly.

## 4. Discussion

This study aimed to describe pain, disability and deep abdominal muscle activation from week to week during tailored exercises for pregnancy-related lumbopelvic pain. The results suggested that the exercise program lead to reduced pain and disability for all sixteen participating women with persisting lumbopelvic pain after delivery. However, there were considerable intra-subject variations in symptoms over the baseline periods as well as over the intervention period, resulting in an unstable standard of comparison. There were no consistent patterns in the voluntary activation of transversus abdominis as measured by lateral slide. Therefore, this study does not suggest that improved voluntary activation of transversus abdominis is related to symptom reduction for women with persistent pelvic pain after delivery.

It might be challenging to distinguish the natural course of improvement due to normalized hormonal levels and weight, from treatment-induced improvement. There is considerable spontaneous improvement in lumbopelvic pain during the first weeks after delivery. For instance, in a large randomized trial, pain resolved within the first 12 weeks after delivery in 99% of the women, irrespective of type of treatment (Elden, Hagberg, Olsen, Ladfors & Ostgaard, 2008). However, when lumbopelvic pain pain persists 3-6 months after delivery it is not likely to resolve spontaneously (Larsen et al., 1999) and therefore improvement for participants in the present study was not likely spontaneous.

A recent systematic review concluded that the evidence regarding effectiveness of physiotherapy for low back and/or pelvic pain after delivery is inconclusive (Ferreira, & Alburquerque-Sendin, 2012). This review suggested that individually instructed exercises seem to be more promising than exercises performed without guidance. The most convincing evidence stems from a study where 20 weeks with rehabilitation including specific motor control exercises was superior to 20 weeks with individualized physiotherapy without motor control exercises in the treatment of 81 women with pelvic girdle pain after pregnancy (Stuge, Veierod, Laerum, & Vollestad, 2004). Exercises were performed without pain and included body awareness in daily activities as well as activation of global stabilizing muscles and of transversely oriented abdominal muscles (Stuge, Laerum, Kirkesola, & Vollestad, 2004). The chosen exercises aimed to affect both the local and the global stability system, and whether one system had a stronger influence on improvement than the other is unknown. The present study supports the observation that individually instructed exercises with ergonomic advice leads to reduced symptoms. We aimed to scrutinize the effect of transversus activation, in order to distinguish the effect of local abdominal muscle control. The deep local muscles transversus abdominis, m. multifidus and pelvic floor muscles are believed to stabilize the pelvic- and low back articulations (Kibler, Press, & Sciascia, 2006; Pel, Spoor, Pool-Goudzwaard, Hoek van Dijke, & Snijders, 2008) combined with global stabilisation from more superficially situated muscle-tendon-fascia-slings (Vleeming, Stoeckart, Volkers, & Snijders, 1990). Transversus abdominis form a belt around the anterolateral part of the abdomen with its horizontal fibres. The muscle is broad with attachments to the lumbar vertebrae via the thoracolumbar fascia, and to the pelvis and rib cage. It crosses the sacroiliac joints and clamps the sacrum between the coxal bones (Pel et al., 2008). During an abdominal drawing-in exercise, transversus abdominis contracts bilaterally and forms a musculofascial band that tightens like a corset (Hides et al., 2006). This deep muscle is assumed to contribute to stability in three ways; by regulating intra-abdominal pressure (Cresswell, Grundstrom, & Thorstensson, 1992; Hodges et al., 2003), by transmitting force to the lumbar spine via the thoracolumbar fascia (Barker et al., 2006), and by increasing sacroiliac joint stiffness (Richardson et al., 2002). It’s activation has been observed to correlate with low back pain (Gildea, Hides, & Hodges, 2013) and to serve as a biomarker for motor control problems associated with longlasting pain in the lumbopelvic region (Hodges, & Tucker, 2011).

However, to date it is unclear whether activation of transversus abdominis should be viewed more like a passive bystander / biomarker than as a contributing cause of chronic low back pain (Hodges, & Tucker, 2011). Results from this study did not suggest that improved voluntary transversus abdominis activation go hand in hand with symptom reduction in women with pregnancy-related lumbopelvic pain. In pregnancy-related pelvic and low back pain, increased laxity of ligaments may lead to more reliance of fine-tuned local muscle activity to obtain ideal weight transfer in the lumbo-pelvic region. Ligament laxity, however, probably reaches normal levels during the first months post partum and consequently was not a main issue in the current study population. Specifically targeting the deep muscles to compensate for reduced support from the passive tissues may be more relevant in- and immediately after pregnancy, when the pregnancy hormones changes tissue quality. After the immediate post partum period, other factors may be more important. In line with this, no differences were observed between 12 women with pelvic girdle pain and 8 women who had recovered from pelvic girdle pain in activation of deep abdominal muscles in a previous study (Stuge, Mørkved, Dahl, & Vollestad, 2006). The present study is in line with this: the data gives no support for the importance of isolated transversus activation as measured by slide in supine position.

Motor control in a broader sense was also targeted though not registered in the present study. During Swiss ball exercises, participants were dependent on intrinsic feedback on whether the neutral spine position was maintained. The intrinsic feedback may be more transferable to everyday activities than the feedback on muscle performance provided by isolated deep muscle activation guided by ultrasound. In everyday activities, motor control depends on intrinsic feedback during simultaneous and coordinated use of the deep and superficial muscles. By instructing the participants to keep a neutral spine while performing demanding exercises, the aim of the Swiss ball exercises was to stimulate muscular capacity and motor control simultaneously. Around the neutral position, the spine exhibits the least stiffness (Panjabi, 1992b) and exercising in this position has been advocated (Richardson, Hodges, & Hides, 2004). It has been suggested that the combination of unstable and decreased base of support also induce increased activation of the deep abdominal muscles. The deepest abdominal muscles automatically increased their activation when the base of support was decreased by raising one foot off the floor while sitting on a gym ball in 30 healthy young adults (Ainscough-Potts, Morrissey, & Critchley, 2006). Merely sitting on a gym ball, however, did not increase deep muscle activation compared to relaxed sitting on a chair. Performing curl-ups on a labile surface approximately doubled abdominal muscle activity registered by surface-EMG over superficial and mid-layer abdominal muscles (Vera-Garcia, Grenier, & McGill, 2000). A recent study indicates that the labile surface increased the activity in lumbar multifidus in Swiss ball exercises compared to a stable surface (Scott, Vaughan, & Hall, 2015). The mix of specific low-load motor control exercises and higher load unstable exercises might have increased the effect of exercises by trigging combined global and local muscle activation.

Diastasis of the rectus abdomins with a midline separation along the linea alba is prevalent in late pregnancy and still for 35-40 % of women six months after delivery (Fernandes da Mota, Pascoal, Carita, & Bo, 2015). It has been suggested that since the ability to stabilize the pelvis against resistance is reduced, abdominal muscle exercises should be chosen with care in the immediate post partum period (Gilleard, & Brown, 1996). However, half a year after childbirth, the prevalence of lumbopelvic pain was similar for women with and without diastasis of the rectus abdominis muscles in 84 Portuguese women (Fernandes da Mota et al., 2015). Again, pregnancy-related changes in tissue quality were most likely not directly connected to symptoms in the present study. On the other hand, diastasis along linea alba may weaken the validity of transverses abdominis slide as an expression for deep abdominal muscle activation since the relation between frontal plane movement of the myofascial junction and neuromuscular recruitment may be changed when the fascial tissue offers less resistance. Additionally, two of the participants in the present study had undergone caesarean sectio 3 months before inclusion (Table 1), which may also affect the way the abdominal muscles are activated.

A clinical implication may be that isolated exercises for transversus abdominis is not essential for patients with lumbopelvic pain after pregnancy. However, the exploratory design call for cautious interpretation of results. For instance, considering the broad targeting of the exercises, the lack of correlation between symptoms and muscle activation may be caused by a non-functional method to register muscle activation in the present study. Distribution of synergetic muscle coordination during weight-bearing tasks may be a better way to estimate motor regulations and movement control and new methods for fine-tuned registration of muscle activation during movements are under development. Additionally, the treatment goals were multifaceted, and other factors, for instance increased self-efficacy, might have mediated symptom reduction. Consequently, results need to be replicated in controlled studies in order to be generalizable.

## 5. Conclusion

In this study, women with lumbopelvic pain after delivery reported reduced pain and disability after an individually adjusted exercise intervention. Nine out of the 15 participants obtained clinically significant pain reduction over the intervention period. Seven patients obtained clinically significant reduction of disability as well as pain. Our data do not suggest that there is a correlation between voluntary activation of transversus abdominis and symptom relief in persistent pregnancy-related lumbopelvic pain.
